# Repeated Tetrahydrocannabinol Injections Induce Tolerance but Do Not Disrupt Ongoing Behaviour Upon Withdrawal in Male and Female Rats With Inflammatory Pain

**DOI:** 10.1002/ejp.70279

**Published:** 2026-04-24

**Authors:** Michael M. Morgan, Christa M. Hickey

**Affiliations:** ^1^ Washington State University Vancouver Vancouver Washington USA

**Keywords:** cannabis, dependence, sex differences, wheel running

## Abstract

**Background:**

Many people use cannabis to self‐medicate for pain. Little is known about the impact of pain on tolerance and spontaneous withdrawal to delta‐9‐tetrahydrocannabinal (THC), the primary psychoactive compound in cannabis. Our previous research with the opioid morphine suggests persistent pain will increase the magnitude and duration of THC withdrawal.

**Methods:**

Male and female Sprague–Dawley rats were individually housed in a cage with a running wheel to provide a continuous and objective measure of well‐being. All rats were injected with Complete Freund's Adjuvant (CFA) into the right hindpaw to induce inflammatory pain. Beginning 1 day later, rats received twice daily THC or vehicle injections for 7 days followed by assessment of tolerance and spontaneous withdrawal.

**Results:**

Administration of CFA decreased wheel running. Twice daily injections of THC (3 mg/kg/injection) caused a further reduction in running in male and female rats. Tolerance to the THC‐induced decrease in running was more pronounced in male compared to female rats. There was no evidence of spontaneous withdrawal to THC despite continuous assessment for 6 days. Likewise, withdrawal had no effect on body weight.

**Conclusion:**

The lack of spontaneous withdrawal in rats with hindpaw inflammation is consistent with our recent study showing a lack of spontaneous withdrawal to THC in pain free rats, but opposite to our opioid research showing enhancement of spontaneous withdrawal to morphine in rats with hindpaw inflammation. In sum, persistent inflammatory pain does not appear to alter the effects of repeated THC injections in male or female rats.

**Significance:**

The use of THC as a treatment for pain is limited by side effects and tolerance but not by dependence associated withdrawal symptoms.

## 1 Introduction

Pain is the primary medical reason people take cannabis (Sexton et al. 2016; Walsh et al. 2013). The antinociceptive effects of delta‐9‐tetrahydrocannabinol (THC), the primary psychoactive compound in cannabis, have also been documented in animal research (Blanton et al. 2021; Martin and Lichtman 1998). Although the antinociceptive effects of THC are mild compared to that produced by opioids (Lötsch et al. 2018), the safety profile is much better. Tolerance and dependence develop rapidly to repeated opioid administration (Bailey and Connor 2005; Bobeck et al. 2012; Kandasamy et al. 2017; Morgan and Bobeck 2025), and opioid side effects and withdrawal are severe (Gowing et al. 2017; Hill and Canals 2022). Although tolerance and withdrawal to THC have been reported, almost nothing is known about tolerance and withdrawal when THC is administered to treat pain.

Tolerance to the antinociceptive effects of THC has been reported in male and female rats with (Craft et al. 2023) and without persistent pain (Marusich et al. 2015; Wakley et al. 2014). Dependence also has been reported, but withdrawal symptoms are mild. Typical withdrawal symptoms in humans consist of irritability, anxiety and insomnia (Budney et al. 2004; Chesney et al. 2024). Precipitation of withdrawal in animals by administration of a cannabinoid receptor antagonist is associated with a range of motor behaviours (e.g., shakes, tremors, licking, arched back) (Aceto et al. 1995; Bruijnzeel et al. 2016; Marusich et al. 2015; Tsou et al. 1995). In addition, anxiety has been reported weeks after precipitated withdrawal in rats made dependent on the cannabinoid receptor agonist WIN55212‐2 (Brewer et al. 2024).

The symptoms of spontaneous THC withdrawal in rats are less pronounced if they occur at all (Aroni et al. 2024; Harte‐Hargrove and Dow‐Edwards 2012). There are two major challenges in assessing spontaneous withdrawal. Are the symptoms the same as in precipitated withdrawal and when do those symptoms occur? Our recent manuscript addressed these questions by assessing the impact of withdrawal on voluntary home cage wheel running for 6 days following termination of THC administration (Hickey et al. 2025). That study found no obvious signs of spontaneous withdrawal in pain‐free male and female rats.

Previous research with opioids shows clear changes in voluntary wheel running during spontaneous withdrawal. Spontaneous withdrawal from morphine or fentanyl causes a decrease in dark phase wheel running that lasts approximately 2–3 days (Morgan, Hilgendorf, and Kandasamy 2023; Morgan and Ataras 2022) and an increase in light phase running that corresponds with insomnia (Morgan et al. 2021; Morgan and Nguyen 2024). The magnitude and duration of the opioid withdrawal‐induced decrease in running were enhanced in rats with inflammatory pain (Kandasamy et al. 2017). The objective of the present experiment was to test the hypothesis that inflammatory pain will have a similar effect on spontaneous withdrawal to THC, essentially producing withdrawal symptoms where none were evident in our previous experiment using pain‐free rats (Hickey et al. 2025).

## 2 Methods

### 2.1 Animals

A total of 32 adult Sprague–Dawley rats (Envigo Laboratories, California), 16 male and 16 female, were used. Rats were housed in same‐sex pairs for 1 week upon arrival and then housed individually in a standard Plexiglas cage fitted with a top‐mounted stainless steel running wheel (Tecniplast, Starr Life Sciences Corp; Pennsylvania). Animals were maintained on a 12:12‐h light–dark cycle (lights off at 10:30 or 11:00 AM depending on the cohort test room) in a temperature and humidity‐controlled room. Food and water were available ad libitum. Cages were changed once per week. This experiment was approved by the Washington State University (WSU) Institutional Animal Care and Use Committee (IACUC) and was conducted in a manner consistent with ARRIVE Guidelines and the *Guide for the Care and Use of Laboratory Animals* (2011).

### 2.2 Wheel Running

Rats had continuous access to a running wheel in the home cage. The wheel had a diameter of 33 cm so each revolution was approximately 1 m. Data were collected in 5‐min bins 24 h a day throughout the experiment. Rats were only removed from the cage for daily body weight measurements, injections and for the weekly cage change. These things occurred just prior to the beginning of the dark phase of the circadian cycle. The only exception was administration of a second injection 6 h into the dark phase for 7 days. No data were analysed during the 2 h preceding the light phase to avoid confounds produced by handling the rats or anticipation of handling.

### 2.3 Experimental Design

**FIGURE 1:**
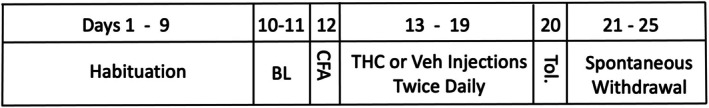
Experimental timeline. The last 2 days of the habituation period were averaged for the baseline value. CFA was injected into the right hindpaw immediately prior to the beginning of Day 12. Half the rats were injected with THC and the other half with vehicle (Veh) twice daily for 7 days. All rats were injected with THC on Day 20 to assess tolerance (Tol). Rats were euthanized after the last day of withdrawal assessment (Day 25).

Rats were allowed to habituate to the running wheel cage for 11 days. The average of the data from the last 2 days was used as the baseline value. Rats were injected with CFA into the right hindpaw immediately prior to the beginning of the dark phase on Day 12. Beginning on Day 13, rats were injected twice daily with THC (3 mg/kg/injection, s.c.) or vehicle (saline:ethanol:cremophor, 18:1:1) for seven consecutive days. Injections were administered 5–10 min prior to the dark phase and again 6 h later. THC was diluted with the vehicle from a solution provided as a gift from the NIDA Drug Supply Program. Tolerance was assessed by injecting every rat with THC (3 mg/kg, s.c.) on the day following the last THC/vehicle injections (Morgan and Bobeck 2025) and measuring the impact on wheel running. Spontaneous withdrawal was assessed continuously starting 1‐day after the tolerance test (Day 21) and ending 6 days after the last THC injection (Day 25). Rats were euthanized with isoflurane following the final day of withdrawal assessment. The timeline is presented in Figure 1.

### 2.4 Statistics

The experiment employed a 2 × 2 factorial design with sex and THC administration as between‐subjects factors (8 rats/condition). Three aspects of wheel running were analysed: (1) Total wheel revolutions during the 12 h dark phase; (2) Total wheel revolutions during the first 10 h of the light phase; and (3) Maximum running speed. Maximum running speed was defined as the highest number of wheel revolutions during a 5‐min bin. Wheel running and body weight data were analysed with 2‐ and 3‐way ANOVAs (Sex × THC × Day) using SPSS. Analysis of sex differences in dark phase wheel running, maximum running speed and body weight required converting data to a percent of baseline because of significant differences in baseline values (Table 1). Light phase running was too low during the baseline period to accurately convert data to a percent of baseline. Data were only collected for the first 10 h of the 12‐h light phase because running levels increased dramatically during the last 2 h of the light phase and data collection was disrupted during the last hour to provide animal care, assess body weight and administer injections. Data were analysed separately for each section of the experiment: Baseline, CFA administration, THC administration, THC tolerance test and spontaneous withdrawal. Two female rats from each group were not tested on the tolerance test so the sample size for that part of the experiment was *N* = 6 instead of 8. Statistical significance was defined as a probability of < 5%.

**TABLE 1:** Sex differences in baseline data (Mean ± SEM).

	Body weight (g)	Dark running	Light running	Maximum speed
Female	185 ± 3.6	7230 ± 986	956 ± 253	208 ± 20
Male	265 ± 3.0	2311 ± 407	289 ± 99	124 ± 11

*Note:* Running is presented as number of wheel revolutions. Maximum speed is the number of revolutions in 5 min.

## 3 Results

### 3.1 Baseline Data

Male and female rats differed on all baseline measures (Table 1). Male rats were significantly heavier than female rats on the last day of the habituation period (*t*_30_ = 16.979, *p* < 0.001). Female rats ran significantly further than male rats during the dark (*t*_30_ = 4.611, *p* < 0.001) and light (*t*_30_ = 2.453, *p* = 0.02) phases of the circadian cycle. In both cases, average male running was approximately 30% of the running in female rats. Female rats also attained a higher maximal speed compared to male rats (*t*_30_ = 3.730, *p* < 0.001).

### 3.2 Hindpaw Inflammation

**FIGURE 2:**
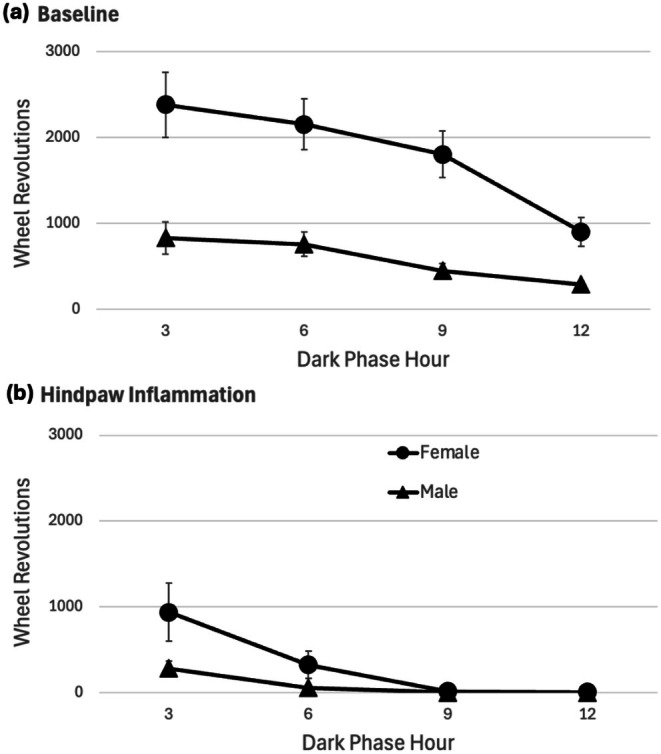
Hindpaw inflammation decreases wheel running across the entire 12‐h dark phase. (a) Baseline wheel running in female rats is greater than in male rats, but both follow the same pattern with running gradually decreasing across the dark phase. (b) CFA administration caused a dramatic decrease in wheel running beginning within the first 3 h of the dark phase until almost all running ceased during the last 6 h of the dark phase.

A mean decrease of 10.2 g of body weight for male rats and 2.6 g for female rats occurred 24 h after the CFA injection. Comparison of this weight loss as a percent of baseline revealed that male rats lost significantly more weight than female rats (96.2% vs. 98.5% of baseline weight, respectively) (*t*_30_ = 3.634, *p* < 0.001). Administration of CFA also caused a profound decrease in wheel running. In the 12‐h dark phase following CFA administration, male rats ran 15.1% ± 3.4% of their baseline value and female rats ran 21.6% ± 5.8% of their baseline value. These decreases in running from baseline to CFA administration resulted in a significant main effect of Day (*F*(1,29) = 49.368, *p* < 0.001). There was no sex difference in this decrease when analysed as a percent change from baseline (*t*_29_ = 0.959, *p* = 0.346). In contrast to the gradual decrease in running that occurs across the dark phase of the circadian cycle during baseline (Figure 2a), wheel running following CFA administration was greatly reduced within the first 3 h and was almost completely abolished during the remainder of the dark phase (Figure 2b). Most rats injected with CFA did not use the running wheel at all during the last 6 h of the dark phase or the subsequent light phase. None of the male rats achieved more than nine revolutions during the entire 10 h light phase measurement following the CFA injection, and 11 of the 16 female rats ran nine revolutions or less during the first 10 h of the light phase.

### 3.3 THC Injections

**FIGURE 3:**
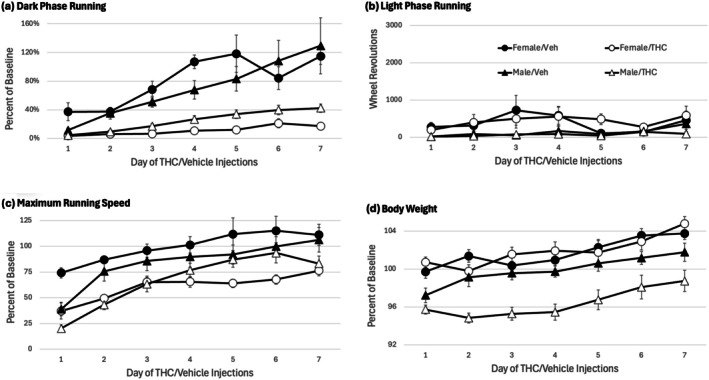
THC injections reduced wheel running and body weight. Injection of THC before and 6 h into the dark phase reduced dark phase wheel running (a), maximum running speed (c), and body weight (d) compared to rats injected with vehicle. The decrease in dark phase running and running speed was comparable in male and female rats, whereas the decrease in body weight was only evident in male rats. Running during the light phase (b) was very low and not altered by THC administration. Given sex differences in baseline values, data in graphs a, c and d are presented as a percent of baseline. Running levels were so low during the light phase (b), data are presented as wheel revolutions, not percent of baseline.

Twice daily THC or vehicle injections began 24 h after the CFA injection. Administration of THC caused a significant decrease in wheel running during the dark phase (Figure 3a) compared to vehicle treated rats (*F*(1,28) = 51.645, *p* < 0.001). Running increased each day as rats recovered from the CFA injection, but this increase was significantly greater in vehicle compared to THC treated rats (THC × Day interaction: *F*(6,168) = 5.797, *p* < 0.001). There was no significant difference in dark phase running between male and female rats (*F*(1,28) = 0.019, *p* = 0.891). Light phase running was very low for the entire 7‐day injection period (Figure 3b). Administration of THC also caused a significant reduction in maximum running speed (Figure 3c) compared to vehicle treated rats (*F*(1,28) = 21.585, *p* < 0.001). This reduction in running speed was comparable in male and female rats as indicated by the lack of a significant effect of Sex (*F*(1,28) = 0.61, *p* = 0.441). Repeated THC administration also caused a significant reduction in body weight (Figure 3d) in male, but not female rats as indicated by a significant THC × Sex interaction (*F*(1,28) =7.601. *p* = 0.01).

### 3.4 THC Tolerance

**FIGURE 4:**
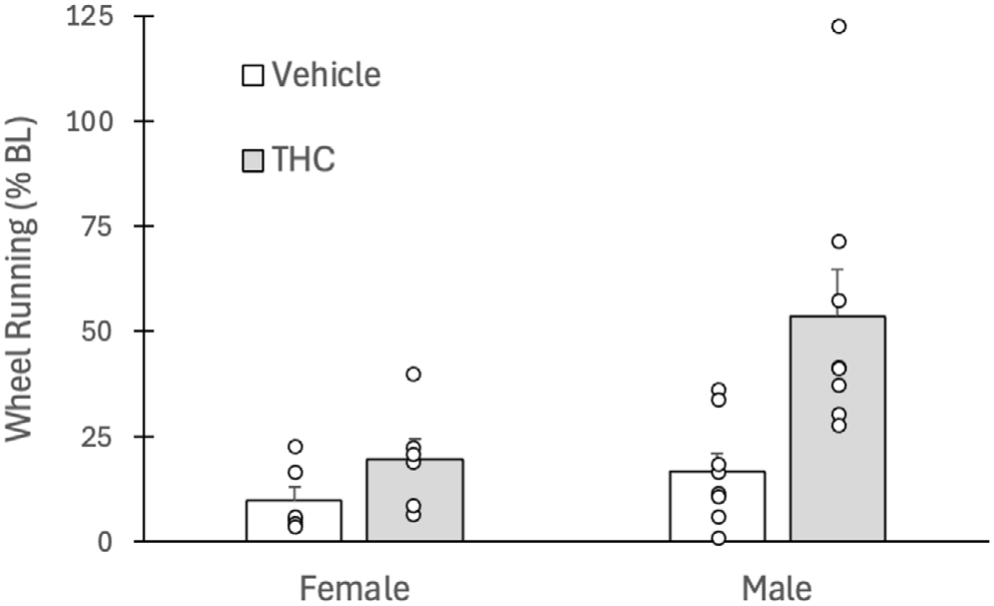
Tolerance to the THC‐induced decrease in wheel running. Administration of THC caused a large decrease in dark phase wheel running in vehicle (rats receiving THC for the first). THC also caused a decrease in dark phase running in rats previously treated with THC twice daily for 7 days but this decrease was blunted by the prior THC injections as would be expected with the development of tolerance. Tolerance, measured as a return to baseline running levels, was more pronounced in male compared to female rats.

Tolerance was assessed by injecting all rats with 3 mg/kg of THC on the day after the last of the seven twice daily THC/vehicle injections. Administration of THC caused a large decrease in dark phase wheel running in vehicle rats receiving THC for the first time (Figure 4). A decrease in running also occurred in rats that had received twice daily injections of THC for 7 days compared to their baseline levels of running. However, administration of THC produced a significantly greater decrease in wheel running in the naïve vehicle rats compared to the THC experienced rats (*F*(1,24) = 9.988, *p* = 0.004). Moreover, male rats displayed greater tolerance than female rats as indicated by a significant main effect of Sex (*F*(1,24) = 7.757, *p* = 0.01).

### 3.5 THC Withdrawal

**FIGURE 5:**
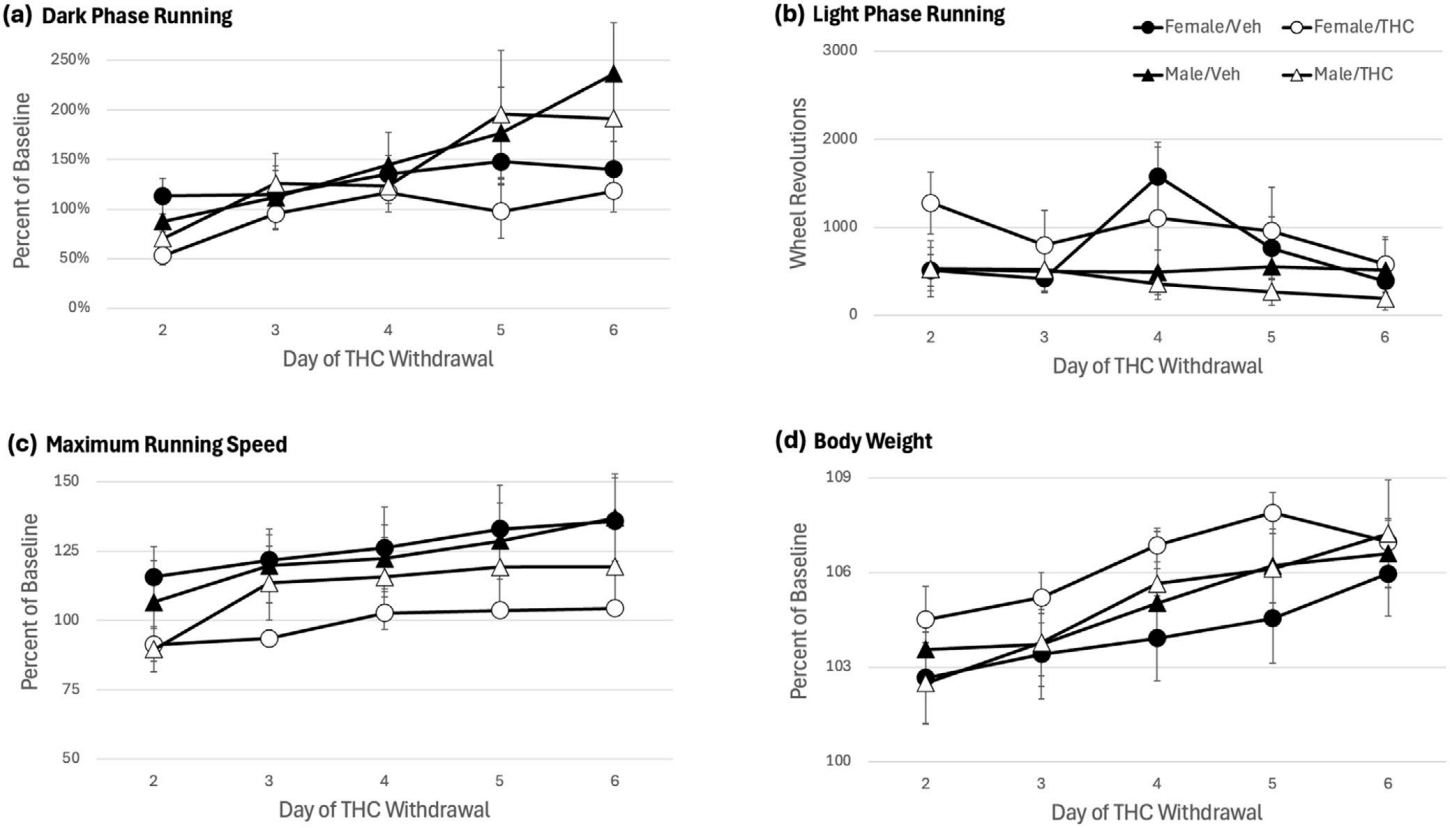
Spontaneous withdrawal from THC had no effect on wheel running or body weight. There was no difference between rats withdrawn from THC compared to vehicle controls on dark phase running (a), light phase running (b), maximum running speed (c) or body weight (d). All data except light phase running are presented as percent change from baseline so male and female rats could be compared. Light phase running was too low to accurately calculate a percent change from baseline for all rats.

Withdrawal was assessed for 6 days following the THC injection for the tolerance test. The tolerance test (Figure 4) shows the data for the 12‐h dark phase immediately following this last THC injection. Figure 5 shows withdrawal Days 2–6. No change in dark phase wheel running is evident in rats withdrawn from THC compared to vehicle (Figure 5a) (*F*(1,28) = 0.764, *p* = 0.390). Although there was a significant increase in dark phase running across days (*F*(4,112) = 12.308, *p* < 0.001), this increase was not related to prior THC treatment as evident by the lack of a significant Day × THC interaction (*F*(4,112) = 0.509, *p* = 0.729). Likewise, there was no significant effect of THC withdrawal on light phase running (Figure 5b; *F*(1,28) = 0.014, *p* = 0.906), maximum running speed (Figure 5c; *F*(1,28) = 2.920, *p* = 0.099), or body weight (Figure 5d; *F*(1,24) = 1.106, *p* = 0.303).

## 4 Discussion

The two primary findings of this manuscript are that administration of 3 mg/kg of THC causes a decrease in wheel running and repeated THC administration produces greater tolerance to the decrease in wheel running in male compared to female rats. There was no evidence for spontaneous withdrawal measured as a change in wheel running or body weight. These data are almost identical to the results of our previous analysis of tolerance and withdrawal to THC in pain‐free rats and indicate that pain does not alter tolerance or dependence to THC (Hickey et al. 2025).

The finding that baseline voluntary wheel running is greater in female compared to male rats is well‐known (Carrera et al. 2011; Titchenal 1988). The present study shows that male Sprague–Dawley rats run about 30% as much as female rats during both the dark and light phases of the circadian cycle. Likewise, the large decrease in wheel running caused by injection of CFA into the hindpaw has been reported previously (Kandasamy et al. 2016). The present study shows a near complete inhibition of wheel running beginning approximately 6 h after the injection and persisting through the light phase before rats begin the gradual day‐by‐day recovery of running.

Antinociceptive effects of THC have been shown to occur using acute pain tests whether rats have inflammatory pain (Britch et al. 2020; Craft et al. 2023) or not (Lichtman and Martin 1991; Tseng and Craft 2001). However, caution must be used in interpreting these results because many of these studies use large doses of THC (≥ 3 mg/kg) known to inhibit movement (Wakley and Craft 2011; Wiley and Burston 2014). When nociception is assessed using pain‐depressed tests such as wheel running (Kandasamy and Morgan 2021), relatively mild antinociceptive effects occur and only at a dose of 0.32 mg/kg (Kandasamy et al. 2018; Morgan, Stickney, and Wilson‐Poe 2023). Given that THC is known to reduce inflammatory pain (Britch et al. 2020; Craft et al. 2013, 2023), there is little doubt the 3 mg/kg dose used in the present study produced antinociception, but this antinociception did not restore wheel running because of the direct inhibitory effect on locomotion. Our previous research shows that increasing the dose of THC from 0.32 mg/kg to 0.56 or 1 mg/kg is enough to depress wheel running (Kandasamy et al. 2018; Morgan, Stickney, and Wilson‐Poe 2023). A major advantage of pain‐depressed (e.g., wheel running) as opposed to pain‐evoked (e.g., von Frey, hot‐plate) tests is the ability to distinguish between doses that produce antinociception independent of disruptive side effects.

The decrease in wheel running caused by THC administration in the present experiment is consistent with our previous research showing a pronounced and prolonged decrease in wheel running in pain‐free rats (Hickey et al. 2025). Although wheel running was already reduced by hindpaw inflammation in the present study, administration of THC caused a further decrease in running. The decrease in running caused by THC was comparable in male and female rats and consistent with previous research in which locomotion was assessed in an open field or the rat's home cage (Britch and Craft 2023; Harte‐Hargrove and Dow‐Edwards 2012; Wakley and Craft 2011). Moreover, THC‐induced suppression of locomotion occurs at doses significantly lower than needed to induce catalepsy (Wakley and Craft 2011).

Tolerance to the antinociceptive effects of THC has been documented in both human (Cuttler et al. 2020) and animal (Craft et al. 2023; Wakley et al. 2014) research. Animal research tends to focus on tolerance to the antinociceptive effects of THC. These studies reveal greater tolerance in pain‐free female compared to male rats (Wakley et al. 2014). Tolerance to the antinociceptive effect of THC was less pronounced in rats with hindpaw inflammation and no sex difference was evident in these rats (Britch et al. 2020; Craft et al. 2023). Likewise, there does not appear to be a sex difference in tolerance to THC‐induced locomotor suppression when measured with wheel running (Hickey et al. 2025). The only effect of hindpaw inflammation was to limit the development of tolerance to locomotor suppression in female rats. The present data show that the one situation where tolerance may be greater in male than female rats is suppression of wheel running in rats with hindpaw inflammation. Although the smaller sample size in female rats tested for tolerance (*N* = 6 vs. *N* = 8 for males) makes the female data less reliable than the male data, these sample sizes were sufficient to result in statistically significant sex difference in tolerance.

The THC injection for the tolerance test decreased wheel running for the entire day so assessment of withdrawal‐induced changes in running began 24 h after this injection. Although withdrawal was assessed continuously using wheel running from the second to sixth day following the last THC injection, no significant changes were evident. Female rats undergoing withdrawal tended to have a lower maximal running speed than female controls or male rats, but that was the only potential change in wheel running. In contrast, withdrawal following twice daily injections of the opioid morphine for 5 days caused a large decrease in dark phase wheel running and an increase in light phase running in male and female rats (Morgan et al. 2021; Morgan and Nguyen 2024). Moreover, hindpaw inflammation enhanced the magnitude and duration of the decrease in wheel running in male rats undergoing morphine withdrawal (Kandasamy et al. 2017). The morphine data show that wheel running is a sensitive measure of withdrawal.

The present data indicate that spontaneous withdrawal from THC is mild if evident at all following twice daily injections for 7 days. Previous studies in rats also failed to find statistically significant withdrawal symptoms following discontinuation of much larger doses of THC (> 10 mg/kg/day) (Aceto et al. 1996; Aroni et al. 2024), although mild symptoms have been reported in mice (Kesner et al. 2022; Trexler et al. 2018, 2019). Our data assessing withdrawal continuously using home cage wheel running is consistent with this lack of obvious signs of withdrawal and shows that the lack of withdrawal is not caused by assessment at the wrong time.

In contrast, precipitation of THC withdrawal by administration of a cannabinoid receptor antagonist such as rimonabant produces clear withdrawal symptoms in rats (Aceto et al. 1995; Bruijnzeel et al. 2016; Marusich et al. 2015; Tsou et al. 1995). The problem is that the clinical relevance of studies in which withdrawal is precipitated can be questioned because humans using cannabis undergo spontaneous, not precipitated, withdrawal. Our previous study in pain‐free rats also failed to find consistent changes in wheel running following spontaneous withdrawal from THC (Hickey et al. 2025). The present results extend this finding by showing that even when THC is used to treat pain, spontaneous withdrawal symptoms do not seem to be a problem. The problem of tolerance and dependence is even less likely with a lower antinociceptive dose. The 3 mg/kg dose was used in the present study to ensure a behavioural effect but is 10 times greater than that needed to produce antinociception in rats (Kandasamy et al. 2018; Morgan, Stickney, and Wilson‐Poe 2023).

## Author Contributions

This experiment was conceived and designed by C.M.H. and M.M.M. Data collection was overseen by C.M.H. Data were compiled and analysed by M.M.M. The manuscript was written by M.M.M. and edited by C.M.H. Both authors have approved the final version of the manuscript and agree to be accountable for all aspects of the work.

## Funding

Washington State Alcohol and Drug Abuse Research Program.

## Disclosure

No AI tool is generated.

## Conflicts of Interest

The authors declare no conflicts of interest.
